# Structure of Nanotubes Self-Assembled from a Monoamide Organogelator

**DOI:** 10.3390/ijms21144960

**Published:** 2020-07-14

**Authors:** Samuel Zapién-Castillo, Nancy P. Díaz-Zavala, José A. Melo-Banda, Duncan Schwaller, Jean-Philippe Lamps, Marc Schmutz, Jérôme Combet, Philippe J. Mésini

**Affiliations:** 1 Centro de Investigación en Petroquímica, Tecnológico Nacional de México-Instituto Tecnológico de Ciudad Madero, Prolongación Bahía de Aldair, Ave. de las Bahías, Parque de la Pequeña y Mediana Industria, Altamira 89600, Mexico; samuel.zapien@iest.edu.mx (S.Z.-C.); aaron.melo@itcm.edu.mx (J.A.M.-B.); 2 Institut Charles Sadron, Université de Strasbourg, CNRS, 23 rue du Loess, F-67000 Strasbourg, France; duncan.schwaller@etu.unistra.fr (D.S.); jean-philippe.lamps@ics-cnrs.unistra.fr (J.-P.L.); marc.schmutz@ics-cnrs.unistra.fr (M.S.); jerome.combet@ics-cnrs.unistra.fr (J.C.); 3 International Center for Frontier Research in Chemistry, 8 allée Gaspard Monge, 67000 Strasbourg, France

**Keywords:** nanotubes, monoamide, self-assembly, organogelators

## Abstract

Some organic compounds are known to self-assemble into nanotubes in solutions, but the packing of the molecules into the walls of the tubes is known only in a very few cases. Herein, we study two compounds forming nanotubes in alkanes. They bear a secondary alkanamide chain linked to a benzoic acid propyl ester (HUB-3) or to a butyl ester (HUB-4). They gel alkanes for concentrations above 0.2 wt.%. The structures of these gels, studied by freeze fracture electron microscopy, exhibit nanotubes: for HUB-3 their external diameters are polydisperse with a mean value of 33.3 nm; for HUB-4, they are less disperse with a mean value of 25.6 nm. The structure of the gel was investigated by small- and wide-angle X-ray scattering. The evolution of the intensities show that the tubes are metastable and transit slowly toward crystals. The intensities of the tubes of HUB-4 feature up to six oscillations. The shape of the intensities proves the tubular structure of the aggregates, and gives a measurement of 20.6 nm for the outer diameters and 11.0 nm for the inner diameters. It also shows that the electron density in the wall of the tubes is heterogeneous and is well described by a model with three layers.

## 1. Introduction

Some molecules have the ability to self-assemble into nanotubes by noncovalent bonds. These tubes have attracted much attention for their applications and for a better understanding and control of the formation [1,2,3]. Most self-assembled organic nanotubes were not engineered by a controlled supramolecular design but were discovered by serendipity. Indeed, the wall is constituted of one or a few layers of molecules and the diameter is one to two orders of magnitude higher than the thickness of the wall. Therefore, if one can explain the formation of the layers by noncovalent bonds, the winding of this layer at a larger scale is more complex and involves the asymmetry and the directionality of the interactions. Theoretical work has modeled the formation of nano and microtubes [4,5,6,7,8,9,10,11]. In these approaches, chirality plays a major role: chiral interactions introduce a bending direction leading to a helical folding and ultimately the formation of the tubes. It is supported experimentally by gemini surfactants that form helical tapes with increasing curvature when the rate of chiral counterions increases [12]. If most of the molecules forming nanotubes are chiral, a few are not [13,14,15,16]. Such compounds are of interest because they challenge the models, that need to introduce spontaneous chirality into the aggregates [11]. In counterpart of these theoretical models, the experimental data are scanty. The structures of tubular self-assembly have been resolved only in a few cases by diffraction [17,18]. Even less resolved structural studies are also rare. Transmission electron microscopy often proves the tubular structure; but only elaborate cryotechniques afford neat pictures of the inner structure of the wall of the tubes, and only if it comprises layers of differently marked contrasts [15]. Small-angle scattering techniques are often more efficient to unveil this inner structure, provided the nanotubes have well-defined diameters. Herein, we report an example of an achiral compound able to form nanotubes. We present their gelation properties and structural studies by electron microscopy and small- and wide-angle X-ray scattering (SAXS and WAXS).

## 2. Results

### 2.1. Solubility and Gelation

**Figure 1:**
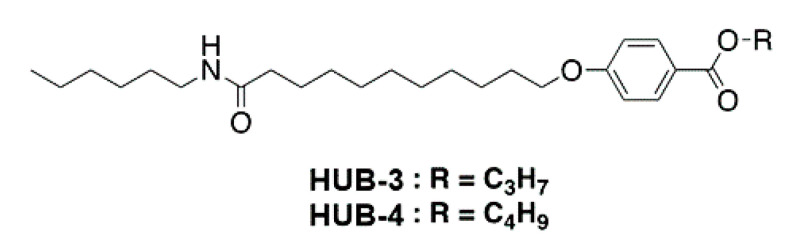
Chemical structure of HUB-3 and HUB-4.

The structure of the studied compounds is depicted in Figure 1.

**Figure 2:**
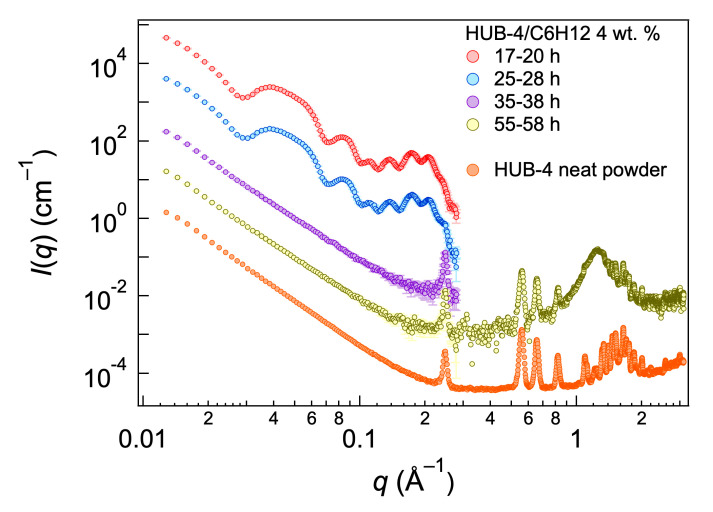
Small-angle X-ray scattering (SAXS) intensities of a HUB-4/C_6_H_12_ gel (*c* = 4 wt.%) at different times, and intensities of neat HUB-4. Between 38 and 55 h, the intensities are the same as the curve for the 35−38 h interval. For clarity, each curve is shifted from the previous one by a factor of 10.

Both HUB-3 and HUB-4 are soluble in CHCl_3_, EtOH and THF. They are insoluble in n-hexane, cyclohexane and *trans*-decaline at room temperature. In these solvents they dissolve upon heating and a gel forms upon cooling for concentrations above 0.2 wt.%. The gels are stable for about 24 h, and they start to become more turbid and eventually precipitate. Such phase transformation has been observed for organogelators. This transformation is visible in the evolution of the scattered intensities with time. Scattered intensities were regularly recorded for HUB-4 during 58 h (3 h measurements) after gel preparation (Figure 2). The curves showed the same intensities for the first 28 h, with many oscillations below 0.3 Å^−1^. Afterwards, the curves switched to a *q*^−4^ curve with a peak at 0.25 Å^−1^. The *q*^−4^ dependency was in agreement with the formation of large objects (Porod scattering) and with the increasing opacity of the samples. At the end of the experiment, the WAXS intensities were also measured and showed a series of Bragg peaks in addition to the one observed below 0.3 Å^−1^, showing the formation of crystals. The final spectra were compared with the spectra of neat powder of HUB-4, and showed the same Bragg peaks. These results showed that HUB-4 formed metastable gels and that the aggregates of these gels transformed slowly back into the crystalline HUB-4.

**Figure 3:**
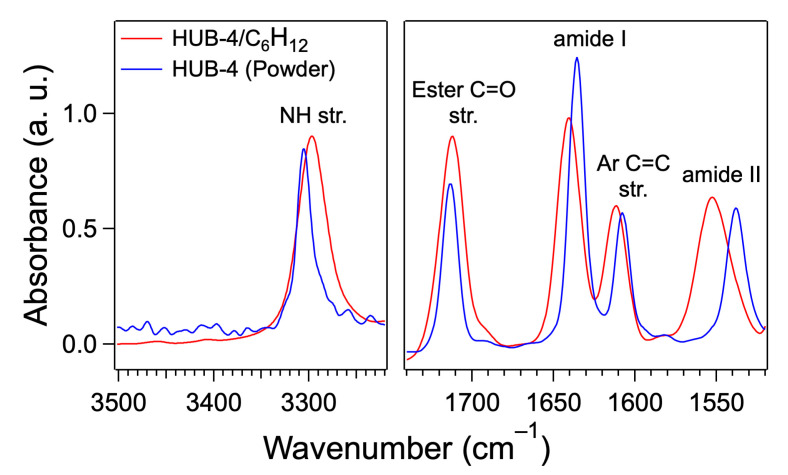
FTIR spectra of HUB-4/C_6_H_12_ (*c* = 4 wt.%) and pure HUB-4 (powder) in the NH and CO stretching area. In both samples the amide groups are H-bonded with each other, but with different H-bonds.

The intermolecular interaction of HUB-4 in the gel was studied by FTIR (Figure 3).

In the spectra of the gel, the NH area showed a major band located at 3297 cm^−1^, which corresponded to H-bonded amides. Similarly, in the CO stretching area, the amide I and amide II modes had frequencies of 1640 and 1552 cm^−1^, which showed that the amides were H-bonded in a large majority (the band at 1611 cm^−1^ was attributed aromatic C=C stretching mode). A small shoulder could be seen at 1690 cm^−1^ and a very weak peak at 3460 cm^−1^. They corresponded to the NH stretching and amide I modes of the free amide. Their presence, even with weak intensities, was attributed to the negligible yet existing fraction of the organogelator in the liquid phase in equilibrium with the solid network of the gel. In the spectrum of pure HUB-4 as a powder the frequencies of the amide bands were also characteristic of H-bonded groups: 3306, 1636 and 1538 cm^−1^ for the NH stretching, amide I and amide II modes, respectively. However, they were shifted to lower frequencies than in the gels, which indicated different H-bonds in the powder. The aggregates in the gel and the powder corresponded therefore to different molecular packing.

### 2.2. Structural Studies by Electron Microscopy

**Figure 4:**
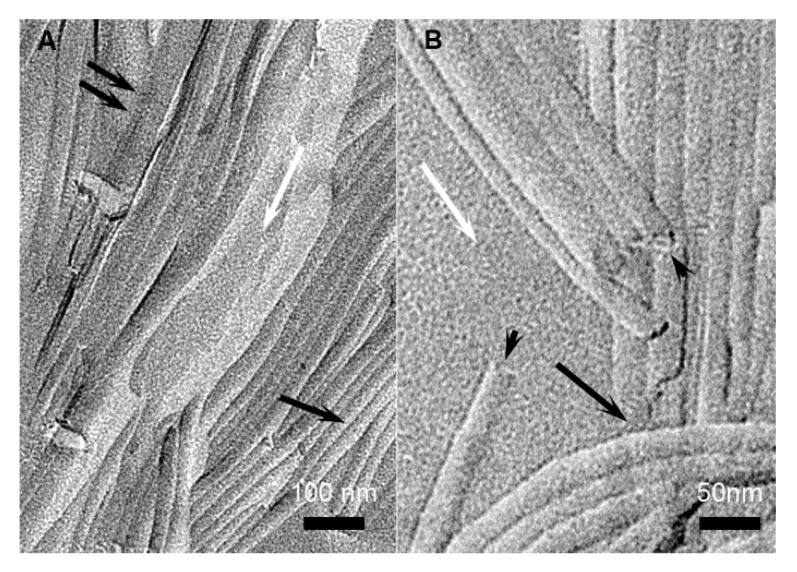
Freeze fractures of gels of HUB-3 in cyclohexane (2 wt.%). (**A**) large field view; (**B**) enlargement showing the ends of tubes. Black arrows: tubes; arrow heads: ends of tubes; white arrows: amorphous solvent; double arrow: lamellar aggregates.

The structures of the gels were observed by TEM. The samples were processed by freeze fracture. The technique consists in a rapid freeze of the samples that kept them in the native state with the solvent amorphous [19,20,21]. It allows the observation of the structures in their surrounding solvent, without concentration change or drying effects. The gels of HUB-3 (Figure 4) were composed of high aspect ratio cylindrical objects with polydisperse diameters of 33.3 ± 9.5 nm. In some fractures, the ends of these objects were visible, and the shadowing clearly showed that they were hollow (Figure 4, arrow heads). The formed objects were not plain cylinders but tubes. Along with those objects, the gels contained less defined structures, including lamellar stacks with a repeat distance of 7−8 nm.

**Figure 5:**
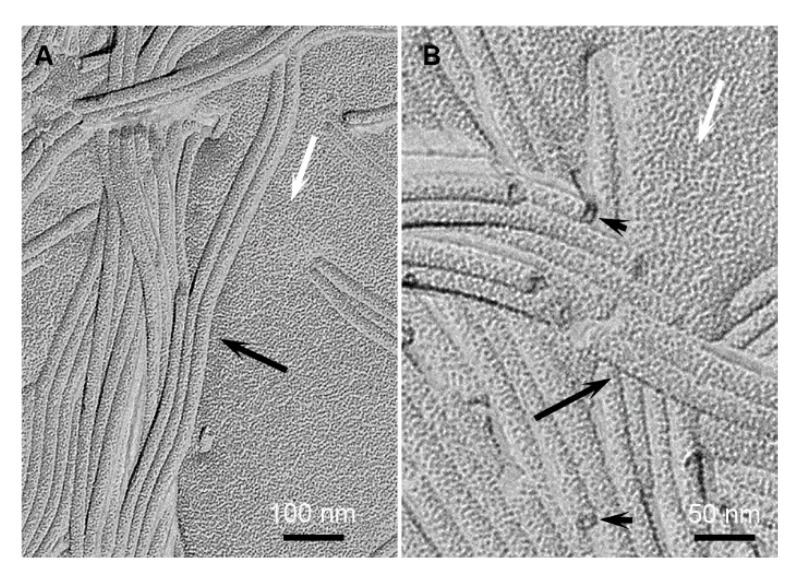
Freeze fractures of gels of HUB-4 in cyclohexane (2 wt.%). (**A**) large field view; (**B**) enlargement showing the end of tubes. Black arrows: tubes; arrow heads: ends of tubes; white arrows: amorphous solvent.

The gels of HUB-4 were composed of tubes of several micrometer lengths and diameters of 25.6 ± 3.3 nm (Figure 5). The tubular shape was also proved by the visible ends of some of the objects, showing a hollow area in the center (Figure 5, arrow heads). The diameters of the tubes had a narrower distribution for HUB-4 than HUB-3. The apparent deviation of 3.3 nm was higher than the real deviation. Indeed, this value amounted to the error inherent to the technique of freeze fracture. It was due to the different embedment depth and to the size of the shadowing particles; and it had the same value for the measured diameters of other tubes with low polydispersity. In the gels of HUB-4, no other objects than tubes were observed. In conclusion, both gelators formed nanotubes of a few tens of nm in diameters. HUB-4 formed only well-defined tubes; HUB-3 formed tubes with polydisperse diameters, along with other types of aggregates.

### 2.3. Structural Studies by SAXS

**Figure 6:**
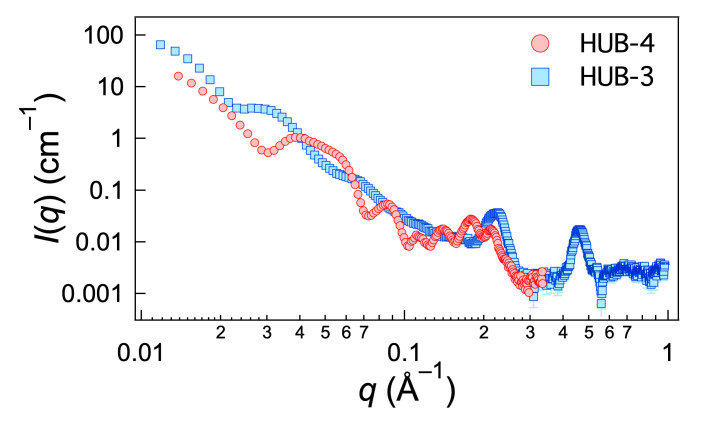
Intensities scattered by gels of HUB-3 and HUB-4 in cyclohexane (2 wt.%).

The gels of HUB-3 and HUB-4 were subjected to X-ray beam, and their scattered intensities measured between 0.01 Å^−1^ and 3 Å^−1^. The measured intensities are shown in Figure 6. The intensity scattered by HUB-4 had sharp features: two oscillations below 0.1 Å^−1^ and between 0.1 and 0.3 Å^−1^ a well-structured massif of 4 or 5 peaks.

The intensity was fitted first by the form factor of infinitely long tubes with a homogeneous scattering length density.

(1) $$I\left(q\right)=\phi_{V}\frac{4\pi^{2}}{\left(r_{1}^{2}-r_{2}^{2}\right)}\frac{1}{q^{3}}{\left(\rho -\rho_{s}\right)}^{2}{\left[r_{1}J_{1}\left(qr_{1}\right)-r_{2}J_{1}\left(qr_{2}\right)\right]}^{2}$$

where *J*_1_ is the Bessel function of first kind and first order and *ϕ*_v_ the volume fraction of theparticles, *r*_1_ and *r*_2_, the external and internal radiuses. As seen by electron microscopy, most of the tubes were pooled in bundles, parallel and in close contact. This rises to inter particle correlations that are modeled by considering pairs of close and parallel tubes. It amounts to multiplying intensity of Equation (1) by the interference term:

(2) $$1+J_{0}\left(2\alpha qr_{1}\right)$$

where 2*αr_1_* corresponds to the distance between the axis of the tubes: if *α* = 1, the tubes are in contact, for *α* > 1 there is a gap between them. We have shown in previous studies on self-assembled nanotubes, that this factor models successfully the difference at low angles between the form factor of the isolated tubes and the experimental intensities.

A Gaussian polydispersity is also introduced on the inner radius *r*_2_ (Equation (3)) while maintaining the wall thickness constant:

(3) $$g\left(r_{2}\right)=\frac{1}{\Delta r_{2}\sqrt{2\pi }}\exp\left[-\frac{{\left(r_{2}-{\bar{r}}_{2}\right)}^{2}}{2\Delta r_{2}^{2}}\right]$$

**Figure 7:**
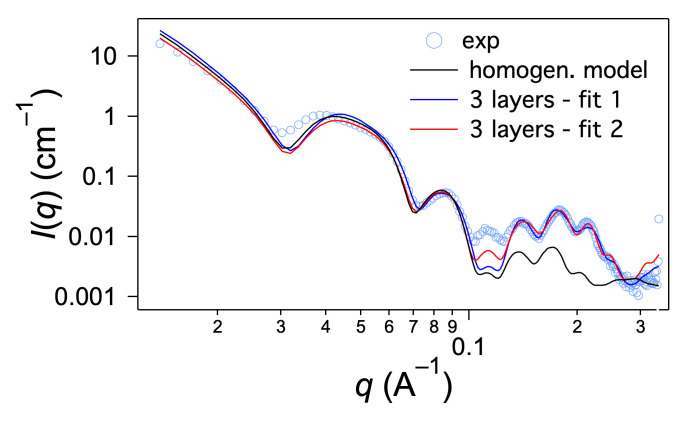
Intensity scattered by HUB-4 (2 wt.% in C_6_H_12_) and fits with the models of homogeneous tube and with the three-layer model. The parameters of the fits 1 and 2 are given in Table 1.

${\bar{r}}_{2}$ is the average inner radius and Δ*r*_2_ is the standard deviation. Finally, a flat background is added to the model and the instrumental resolution is taken into account before comparison with the data. As shown in Figure 7 (homogeneous model), Equations (1)–(3) reproduce the shape of the first oscillations when the outer radius is 10.5 nm and the inner radius 5.5 nm (with *α* = 1 and Δ*r*_2_ = 3 Å); they account also for the oscillations of the massif between 0.1 and 0.3 Å^−1^ but with much lower amplitude. It cannot reproduce the overall shape of the massif. In previous SAXS studies of compounds forming nanotubes [22], we had observed such a massif, although with less marked oscillations: we already had noted it could not be explained by the form factor alone and added an empirical Gaussian contribution to the model.

**Figure 8:**
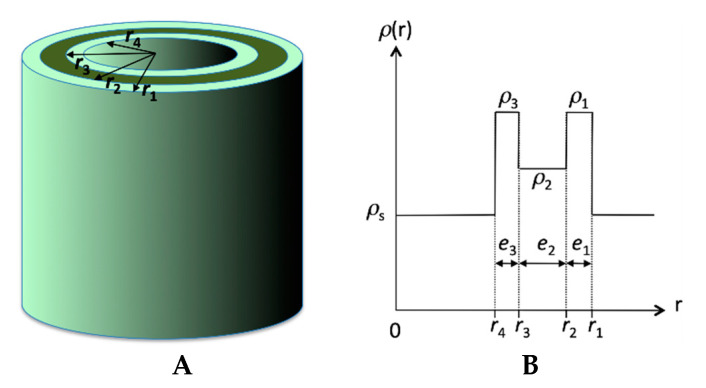
Model of tube with three layers. (**A**) definition of the different radiuses; (**B**) radial scattering length density profile *ρ*(*r*).

In the present case, the structured massif indicated a well-defined tube geometry and molecular organization. Therefore, we applied a form factor of tubes with an inhomogeneous scattering length density along the radial direction reflecting the regular molecular packing. The model was based on a three-shell organization within the wall of the tubes defining a radial scattering length density profile *ρ*(*r*) (Figure 8). The shells are numbered from 1 for the outer one to 3 for the inner one. *ρ*_n_ denotes the scattering length density of the nth layer, and *e*_n_ its thickness. *ρ*_s_ is the scattering length density of the solvent. *r*_n_ is the outer radius of the nth shell and *r*_4_ is the inner radius of the tube. The thicknesses of the layers 1, 2 and 3 are *e*_1_ = *r*_1_ − *r*_2_, *e*_2_ = *r*_2_ − *r*_3_ and *e*_3_ = *r*_3_ − *r*_4_. The total thickness of the tube is *e* = *e*_1_ + *e*_2_ + *e*_3_, and the external radius of the tube is equal to *r*_1_ = *e* + *r*_4_.

The intensity scattered by these tubes is given by Equation (4):

(4) $$I\left(q\right)=\phi_{V}\frac{4\pi^{2}}{\left(r_{1}^{2}-r_{4}^{2}\right)}\frac{1}{q^{3}}{\left[r_{1}\left(\rho_{1}-\rho_{s}\right)\;J_{1}\left(qr_{1}\right)+r_{2}\left(\rho_{2}-\rho_{1}\right)\;J_{1}\left(qr_{2}\right)+r_{3}\left(\rho_{3}-\rho_{2}\right)\;J_{1}\left(qr_{3}\right)+r_{4}\left(\rho_{s}-\rho_{3}\right)\;J_{1}\left(qr_{4}\right)\right]}^{2}$$

This intensity is also multiplied by the structure factor (2) and a Gaussian polydispersity is implemented on the inner radius *r*_4_ while the thickness is kept constant. The experimental intensity was fitted with the theoretical model (Equations (2)–(4)) without additional scale factor. We considered a homogenous gel and fixed the volume fraction *ϕ*_v_ to its theoretical value (0.0159). We only considered the situation where the thicknesses *e*_1_ and *e*_3_ are identical. In the first place, *α* is set at 1 and the scattering lengths density of the outer and inner layers of the cylinders are held equal (*ρ*_1_ = *ρ*_3_). The fitted parameters (*e*_1_, *e*_2_, *r*_4_, *ρ*_1_, *ρ*_2_, Δ*r*_4_, background) are shown in Table 1 (Fit 1).

The model reproduces most of the features of the curve at low angles and at high angles, especially the shape of the first oscillations, and most of the oscillations above 0.1 Å^−1^ (Figure 7). The overall shape of the massif between 0.1 and 0.3 Å^−1^ can therefore be explained by a particular radial scattering length density profile resulting from a regular organization of the molecules within the walls of the tubes.

**Figure 9:**
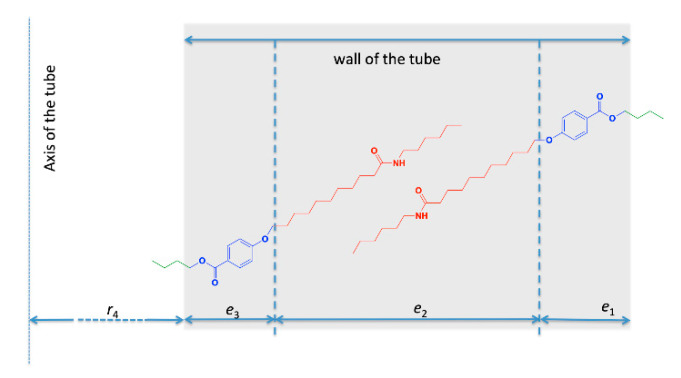
Proposed model for the self-assembly of HUB-4. The tilt angles are arbitrary. The amides are H-bonded with each other.

The fit yields a larger thickness for layer 2 and thinner layers 1 and 3. The scattering length density is lower for layer 2 than layers 1 and 3. The found value *ρ*_1_ and *ρ*_3_ are close to the one expected for the part containing both the aromatic and the ester moieties (see Table 2, entry OC_6_H_4_COOC_4_H_9_). The thickness *e*_2_, of 23 Å is in the register of the length of the amide chain. From these results, we propose that layer 2 contains the amides chains and layers 1 and 3 the aromatic and ester parts (Figure 9). The value of the scattering length density *ρ*_2_ is close to the one expected for close packed amides, but lower. This can be explained by less dense packing in the tubes. However, this layer is not amorphous since the amides are H-bonded with each other as shown above.

Beyond the global agreement with the theoretical description, the oscillation at 0.11 Å^−1^ is underestimated by the fit. The experimental intensity suggests the presence of an additional independent contribution, for instance a Bragg peak in this particular *q*-range. This difference could also originate from a more complex organization that is not correctly described by a simple geometrical model. Furthermore, in previous work, we have shown that with freeze fracture, the diameter was only 3 to 4 nm higher than that observed by small-angle X-ray or neutron scattering [22,24]. This difference was due to the metal particles sputtered to form the replica. This particle layer was about 2 nm in thickness and therefore the apparent diameter in the fractures was increased to twice the layer size. In the present work, the outer diameter found with our model (19.4 nm) was 6 nm lower than that measured by electron microscopy (25.6 nm). This large difference could also reflect the presence of another external layer not directly visible by SAXS and not included in the previous fit. This layer may prevent close contact between the model tubes.

In order to account for this low contrast external layer, we introduced the geometrical parameter *α* (Equation (2)). New fits were performed with independent scattering length densities *ρ*_1_ and *ρ*_3_ and the adjustable *α* parameter. Best results were obtained when the layers 1 and 3 were dissymmetrical, i.e., with different scattering length densities (Figure 2, Fit 2) and when *α* was larger than unity, indicating a gap between tubes. In the previous model, butyl chains were found in shells 1 and 3, i.e., in the external layers of the wall. These chains had a scattering length density close to that of the solvent that made them “invisible” for SAXS. The gap between the tubes should therefore represent the distance filled by these butyl chains. The best fit yielded a shorter thickness for layers 1 and 3, along with higher values for *ρ*_1_ and *ρ*_3_. It was consistent with the fact that in this model the layers 1 and 3 were now filled with a shorter part: only the aromatic part without the butyl ester. The found value for *α* was 1.07, which amounted to an invisible outer layer of 7 Å, which was close to the expected length for the butyl ester (5 Å). With this fit, the overall diameter of the tube, including this outer layer was 2*αr*_1_ and amounted 20.5 nm, a little closer to that found by electron microscopy. Both fits gave therefore, very similar results and converge toward a model for the inner structure of the wall of the tubes (Figure 9).

The intensity scattered by the gels of HUB-3 showed less features than that of HUB-4. Below 0.2 Å^−1^, only attenuated oscillations were visible, consistent with the broad dispersion of diameters of the tubes. These weak oscillations could not be fitted accurately with the model of tubes (even if a large polydispersity was introduced). Above 0.2 Å^−1^ the most salient feature was the presence of two Bragg peaks at 0.22 and 0.45 Å^−1^, hinting at a lamellar organization with a characteristic distance of 29 Å. It was consistent with the observations by TEM, showing lamellar aggregates. These lamellae, as observed on micrographs, had a bigger stacking distance (7–8 nm). This distance was overestimated, both because of the shadowing layer and the tilt angle, but it probably corresponded to twice the distance measured by X-ray. Both the polydispersity of the diameters and the presence of objects of different shapes precluded the fit of these curves.

## 3. Discussion

We have shown that a simple monoamide is an organogelator of alkanes and self-assemble into nanotubes in these solvents. This compound is not chiral and provides another example of a nonchiral compound forming nanotubes. As explained in the introduction, the theoretical models rely on chirality. As shown by some models [11], even if the molecules are achiral it can assemble in a chiral way, if the molecules pack in a chiral way. We have shown that the tubes are metastable, and they transform into crystals after 28 h. Such transformations in organogels, have been observed for other gelators [25,26]. Given the shape of the aggregates, it is relevant to compare HUB-4 also with lipid compounds self-assembling in metastable nanotubes [27]. As Gubitosi et al. have shown lately, kinetically stable tubes formed by lithocholic acid have been proved to be thermodynamically metastable [28]. Like these authors did, we will devote our next studies to map the *c*-*T* phase diagram of these polymorphs.

The structural studies by SAXS clearly showed that the wall of the cylinder was not homogeneous, but had different layers. In this paper and in previous structural studies, the model of the homogeneous tube could not account the number, the location and the intensities of the oscillations between 0.1 and 0.3 Å^−1^. If this simple model gives a good estimation of the outer diameter, an empirical contribution needs to be introduced to reproduce the overall scattering profile. The success of this three-layer model is owed to the intrinsic properties of the tubes: the low polydispersity of their diameters preserves the resolution of the oscillations in this *q*-range. HUB-3 illustrates how polydispersity blurs or dampen the oscillations.

The three-layer model proposed in this paper improves the knowledge of the structure of the tube. It allows the proposition of a model of self-assembly coherent with the molecular parameters and the H-bonds between the amide groups. In this assembly, the amides are in the middle layer and the ester groups in contact with the solvent. This relative orientation is coherent with the solubility of the different parts of the molecule. The esters and aromatic compounds are fully miscible with alkanes, but *N*-alkyl amides and alkanes show a liquid-liquid phase separation [29]. As we have shown, an amide containing gelator, leads to such a liquid-liquid phase separation in the sol [30]. The location of the ester chains is supported by the fact that the parameters of the fits provide an almost invisible outer and inner layer. This will be further explored by SANS, where the contrast with the solvent is higher. The model will be also consolidated by the same studies of the analogues with deuterated ester chains.

## 4. Materials and Methods

### 4.1. Compounds

**Figure 10:**
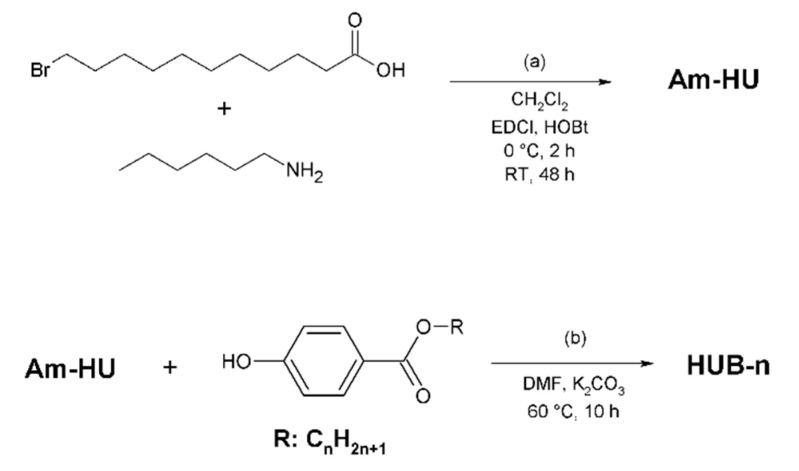
Synthesis of (**a**) bromoamide Am-HU, (**b**) products HUB-3 and HUB-4 (*n* = 3, 4).

HUB-3 and HUB-4 (Figure 1) were synthesized according to Figure 10.

The bromoamide Am-HU was formed by coupling 11-bromoundecanoic acid and n-hexylamine. The corresponding phenols were etherified with Am-HU with fair yields. The products were obtained at a scale of several grams. The NMR, FTIR and mass spectra of the compounds as well as their GPC elugrams are shown in Figures S1 to S11 in Supplementary Materials.

11-bromoundecanoic acid hexylamide (Am-HU): A solution of 11-bromoundecanoic acid (4.81 g, 18.1 mmol) and 1-hexanamine (1.76 g, 17.4 mmol) in dichloromethane (70 mL) was stirred at 0 °C under N_2_. An excess of *N*-(3-dimethylaminopropyl)-*N*′-ethylcarbodiimide hydrochloride (EDCI, 5.06 g, 26.4 mmol) and 1-hydroxybenzotriazole (HOBt, 4.06 g, 30.0 mmol) were added, then stirred 2 h at 0 °C and 48 h at room temperature. The reaction mixture was washed with aqueous NaHCO_3_ (100 mL, 10% *w*/*w*), evaporated and the residue was purified by chromatography (SiO_2_, hexane/ethyl acetate 50% *v*/*v*) to afford Am-HU as a yellowish solid (4.99 g, 83.2% yield). M. p. 58–60 °C; ^1^H NMR (400 MHz, CDCl_3_/TMS): δ (ppm) 0.88 (t, *J* = 6.8 Hz, 3 H, CH_3_), 1.28 (m, 16 H, CH_2_), 1.41 (m, 2 H, Br(CH_2_)_2_C***H_2_***), 1.48 (m, 2 H, NHCOCH_2_C***H_2_***), 1.61 (m, 2 H, CONHCH_2_C***H_2_***), 1.84 (quint, *J* = 7.2 Hz, 2 H, BrCH_2_C***H_2_***), 2.14 (t, *J* = 7.6 Hz, 2 H, NHCOC***H_2_***), 3.23 (q, *J* = 6.8 Hz, 2 H, CONHC***H_2_***), 3.40 (t, *J* = 6.8 Hz, 2 H, BrCH_2_), 5.43 (s, 1 H, NH). ^13^C NMR (100 MHz, CDCl_3_/TMS) δ (ppm): 14.1 (CONH(CH_2_)_5_***C***H_3_), 22.6 (CONH(CH_2_)_4_***C***H_2_), 25.9 (CONH(CH_2_)_2_***C***H_2_), 26.6 (NHCOCH_2_***C***H_2_), 28.2 (NHCO(CH_2_)_2_***C***H_2_), 28.8 (NHCO(CH_2_)_3_***C***H_2_), 29.31 (NHCO(CH_2_)_4_***C***H_2_), 29.34 (Br(CH_2_)_4_***C***H_2_), 29.39 (Br(CH_2_)_3_***C***H_2_), 29.40 (Br(CH_2_)_2_***C***H_2_), 29.7 (CONHCH_2_***C***H_2_), 31.5 (CONH(CH_2_)_3_***C***H_2_), 32.9 (BrCH_2_***C***H_2_), 34.2 (Br***C***H_2_), 37.0 (NHCO***C***H_2_), 39.6 (CONH***C***H_2_), 173.2 (NH***C***O). GC/MS (ret. time, min) 24.73. FTIR (ATR-diamond) ν_max_ (cm^−1^): 3282 (νN–H), 1637 (amide I), 1553 (amide II), 728 (γ(CH_2_)_n_), 687 (νC–Br). MS (ESI+): *m*/*z* 350.19 [M + H]^+^; analysis: found: C, 59.02; H, 9.89; N, 4.27; calculated for C_17_H_34_BrNO: C, 58.61; H, 9.84; N, 4.02.

4-(11-hexylcarbamoyl-undecanoxy)-benzoic acid propyl ester (HUB-3): A solution of 4-hydroxybenzoic acid propyl ester (564 mg, 3.1 mmol) and K_2_CO_3_ (649 mg, 4.7 mmol) in DMF (90 mL) was stirred under N_2_ at 65 °C for 2 h. compound Am-HU (1.2 g, 3.5 mmol) was added and the mixture stirred for additional 8 h. The reactive mixture was poured into deionized water (300 mL), which resulted in the precipitation of a clear solid that was vacuum filtered and recrystallized from acetonitrile to afford pure HUB-3 as a white solid (998 mg, 71.2% yield): M. p. 60–62 °C; ^1^H NMR (400 MHz, CDCl_3_/TMS): δ (ppm) 0.88 (t, *J* = 6.8 Hz, 3 H, CH_3_), 1.02 (t, *J* = 7.4 Hz, 3 H, COO(CH_2_)_2_C***H_3_***), 1.29 (m, 16 H, CH_2_), 1.47 (m, 4 H, Ar–O(CH_2_)_2_C***H_2_***, NHCOCH_2_C***H_2_***), 1.62 (m, 2 H, CONHCH_2_C***H_2_***), 1.78 (m, 4 H, COOCH_2_C***H_2_,*** Ar–OCH_2_C***H_2_***), 2.15 (t, *J* = 7.5 Hz, 2 H, NHCOC***H_2_***), 3.24 (q, *J* = 6.6 Hz, 2 H, CONHC***H_2_***), 4.00 (t, *J* = 6.6 Hz, 2 H, Ar–OCH_2_), 4.24 (t, *J* = 6.7 Hz, 2 H, COOCH_2_), 5.43 (s, 1 H, NH), 6.90 (d, *J* = 8.8 Hz, 2 H, ArC3–H, ArC5–H), 7.98 (d, *J* = 8.8 Hz, 2 H, C2–H, C6–H). ^13^C NMR (100 MHz, CDCl_3_/TMS) δ (ppm): 10.6 (COO(CH_2_)_2_***C***H_3_), 14.1 (CONH(CH_2_)_5_***C***H_3_), 22.2 (COOCH_2_***C***H_2_), 22.6 (CONH(CH_2_)_4_***C***H_2_), 25.9 (CONH(CH_2_)_2_***C***H_2_), 26.0 (NHCOCH_2_***C***H_2_), 26.6 (NHCO(CH_2_)_2_***C***H_2_), 29.1 (NHCO(CH_2_)_3_***C***H_2_), 29.33 (NHCO(CH_2_)_4_***C***H_2_), 29.36 (ArO(CH_2_)_4_***C***H_2_), 29.38 (ArO(CH_2_)_3_***C***H_2_), 29.4 (ArO(CH_2_)_2_***C***H_2_), 29.5 (Ar-OCH_2_***C***H_2_), 29.7 (CONHCH_2_***C***H_2_), 31.5 (CONH(CH_2_)_3_***C***H_2_), 37.0 (NHCO***C***H_2_), 39.5 (CONH***C***H_2_), 66.3 (COO***C***H_2_), 68.2 (Ar–O***C***H_2_), 114.0 (C3, C5, O–(Ar)C***C***H), 122.3 (Ar–C1), 131.5 (Ar–C2, Ar–C6), 162.9 (Ar–C4), 166.6 (***C***OO), 173.1 (NH***C***O). GC/MS (ret. time, min) 36.64. FTIR (ATR-diamond) ν_max_ (cm^−1^): 3304 (νN–H), 1710 (ester νC=O), 1634 (amide I), 1540 (amide II), 1251 (as νC–O–C), 1015 (sy νC–O–C), 1172 (ester as νC–O), 1106 (ester sy νC–O), 843, 767, 694 (oop Ar C–H). HRMS (ESI+): m/z 448.3435 [M + H]^+^ C_27_H_45_NO_4_ requires 448.3421. Analysis: found: C, 72.64; H, 10.15; N, 3.19; calculated for C_27_H_45_N O_4_: C, 72.44; H, 10.13; N, 3.13.

4-(11-hexylcarbamoyl-undecanoxy)-benzoic acid butyl ester (HUB-4): Same protocol as HUB-3: white solid (53.2% yield). M. p. 75-77 °C; ^1^H NMR (400 MHz, CDCl_3_/TMS): δ (ppm) 0.88 (t, *J* = 6.8 Hz, 3 H, CH_3_), 0.97 (t, *J* = 7.4 Hz, 3 H, COO(CH_2_)_3_C***H_3_***), 1.29 (s, 16 H, CH_2_), 1.47 (m, 6 H, COO(CH_2_)_2_C***H_2_***, ArO(CH_2_)_2_C***H_2_***, NHCOCH_2_C***H_2_***), 1.62 (m, 2 H, CONHCH_2_C***H_2_***), 1.76 (m, 4 H, COOCH_2_C***H_2_,*** ArOCH_2_C***H_2_***), 2.14 (t, *J* = 7.6 Hz, 2 H, NHCOC***H_2_***), 3.24 (q, *J* = 6.6 Hz, 2 H, CONHC***H_2_***), 4.00 (t, *J* = 6.6 Hz, 2 H, ArOCH_2_), 4.29 (t, *J* = 6.6 Hz, 2 H, COOCH_2_), 5.39 (s, 1 H, NH), 6.89 (d, *J* = 8.9 Hz, 2 H, ArC3–H, ArC5–H), 7.97 (d, *J* = 8.9 Hz, 2 H, C2–H, C6–H). ^13^C NMR (100 MHz, CDCl_3_/TMS) δ (ppm): 10.6 (COO(CH_2_)_3_***C***H_3_), 14.1 (CONH(CH_2_)_5_***C***H_3_), 22.2 (COOCH_2_***C***H_2_), 22.6 (CONH(CH_2_)_4_***C***H_2_), 25.9 (CONH(CH_2_)_2_***C***H_2_), 26.0 (NHCOCH_2_***C***H_2_), 26.6 (NHCO(CH_2_)_2_***C***H_2_), 29.1 (NHCO(CH_2_)_3_***C***H_2_), 29.34 (NHCO(CH_2_)_4_***C***H_2_), 29.37 (ArO(CH_2_)_4_***C***H_2_), 29.39 (ArO(CH_2_)_3_***C***H_2_), 29.45 (ArO(CH_2_)_2_***C***H_2_), 29.5 (ArOCH_2_***C***H_2_), 29.7 (CONHCH_2_***C***H_2_), 31.4 (C3, COOCH_2_***C***H_2_), 31.5 (CONH(CH_2_)_3_***C***H_2_), 37.0 (NHCO***C***H_2_), 39.5 (CONH***C***H_2_), 66.3 (COO***C***H_2_), 68.2 (ArO***C***H_2_), 114.0 (ArC3, ArC5), 122.6 (ArC1), 131.5 (ArC2, ArC6), 162.9 (ArC4), 166.6 (***C***OO), 173.1 (NH***C***O). GC/MS (ret. time, min) 36.90. FTIR (ATR-diamond) ν_max_ (cm^−1^): 3305 (νN–H), 1710 (ester νC=O), 1634 (amide I), 1539 (amide II), 1251 (as νC–O–C), 1015 (sy νC–O–C), 1172 (ester as νC–O), 1106 (ester sy νC–O), 843, 767, 694 (oop Ar C–H). HRMS (ESI+): m/z 484.3390 [M + Na]^+^; C_28_H_47_NO_4_ requires 484.3397. Analysis: found: C, 72.89; H, 10.17; N, 3.09. Calculated for C_28_H_47_NO_4_: C, 72.84; H, 10.26; N, 3.03.

For the spectroscopic and structural studies, spectroscopic grade solvents were used. *trans*-Decalin was purified on a column of silica.

### 4.2. Characterisation of the Synthesized Compounds

NMR: The spectra were recorded on a Avance III NMR Ascend 400 from Bruker (Billerica, MA, USA), operating at 400 MHz for ^1^H and 100 MHz for ^13^C experiments.

FTIR: The spectra were recorded on a Perkin Elmer (Waltham, MA, USA) Spectrum 100 equipped with an ATR diamond reflection unit, with a resolution of 4 cm^−1^ and an accumulation of 16 scans.

GC-MS: measurements were performed using a GC-MS Clarus 600 system from Perkin Elmer (Waltham, MA, USA) with a nonpolar 5% phenyl 95% dimethyl polysiloxane cross-bonded column and the mass spectrometer was set for EI at 70 eV.

Melting points: They were measured with a melting point apparatus from Fisher−Johns (Pittsburg, PA, USA), at a heating rate of 2 °C/min.

HRMS (ESI+): High resolution mass spectra were recorded with a microTOF II spectrometer from Bruker Daltonics (Bremen, Germany) operating with an electrospray source.

The elemental analyses of the synthesized compounds were carried out on a Thermo Fisher Scientific (Les Ullis, France) Flash 2000 equipment for the simultaneous analysis of carbon, hydrogen and nitrogen.

### 4.3. FTIR of the Gels

The spectra were recorded with a Vertex 70 spectrometer from Bruker Optics (Ettingen, Germany). The samples were inserted inside a homemade cell with NaCl windows and an optical path of 0.1 mm. The spectra of the solvent were measured in the same conditions. The spectra of the samples were compensated from water with OPUS (Bruker Optics, Ettingen, Germany) and they were subtracted from the solvent.

### 4.4. Electron Microscopy

The gels were freeze fractured and replicas were obtained according to a procedure described previously [22]. The replicas were observed with a FEI Tecnai G2 Sphera (FEI-Thermofisher, Eindhoven, Netherlands) operating at 200 kV and with a FEI Eagle 2k-2k ssCCD camera. The distances were measured with the software AnalySIS (SIS-Olympus, Münster, Germany). The diameters of the objects were measured for about 200 objects.

### 4.5. X-Ray Scattering

Small- and wide-angle scattering experiments have been performed on a Rigaku diffractometer (Rigaku Innovative Technology, Inc., Auburn Hills, USA) using a microfocus rotating anode generator (Micromax-007 HF, Rigaku corporation, Tokyo, Japan) operating at 40 kV and 30 mA. The X-ray beam was monochromatized (CuKα radiation, wavelength *λ* = 1.54 Å) and focused with a confocal Max-Flux Optics (Osmic Inc., Troy, MI, USA) and a three pinholes collimation system. Two different configurations were used to probe scattering vectors *q* from 0.01 to 3 Å^−1^ (*q* is defined as (4π/*λ*sin(*θ*/2)) where *θ* is the scattering angle). The low *q* range was measured with a 2D multiwires detector located at 81 cm from the sample. This configuration investigates *q* values between 0.01 Å^−1^ and 0.33 Å^−1^. The high *q* range was recorded on Fuji imaging plates inserted at 10 cm from the sample position, measuring *q* values between 0.1 Å^−1^ and 3 Å^−1^.

The sample was inserted in a homemade container with two calibrated mica sheets windows one millimeter apart. Scattering patterns were treated according to the usual procedures for isotropic small-angle scattering. Data were radially integrated, and corrected for electronic background, detector efficiency, empty cell scattering, sample transmission and sample thickness. Scattering from the pure solvent was measured separately and subtracted from the sample solution according to its volume fraction. Intensity was converted into absolute scale using calibrated Lupolen (LyondellBasell, London, United Kingdom) as a standard. The scattering vectors *q* values were calibrated with the powder diffraction peaks of silver behenate. The instrumental resolution was determined from the linewidth of the diffraction peaks of a tricosane powder.

The corrected scattered intensity *I*(*q*) which represents the differential cross section per unit volume of the solute, was analyzed by the software developed by National Institute of Standards and Technology Center for Neutron Research [31]. The scattering length densities *ρ* (cm^−2^) in Table 2 are derived from:

(5) $$\rho =\frac{0.282\cdot 10^{-12}}{v}ZN$$

where *Z* is the number of electrons of the scattering part, *N* is Avogadro’s number (mol^−1^) and *v* is the molar volume of the part (cm^3^∙mol^−1^). The molar volumes of the different parts of the compounds were estimated by incremental volumes [23].

## Figures and Tables

**Table 1:** Parameters of the fits with the three-layer model.

Parameters	Fit 1	Fit 2
*e*_1_ (Å)	10.4	6.2
*e*_2_ (Å)	23.1	28.6
*e*_3_ (Å)	10.4	6.2
total wall thickness (Å)	43.9	41.0
*r*_4_ (Å)	53.2	55.0
*ρ*_1_ (× 10^10^ cm^−2^)	11.21	12.81
*ρ*_2_ (× 10^10^ cm^−2^)	8.84	8.65
*ρ*_3_ (× 10^10^ cm^−2^)	11.21	11.85
*ρ*_s_ (× 10^10^ cm^−2^)	7.55	7.55
*α*	1	1.07
Δ*r*_4_ (Å)	3.3	3.4
background (cm^−1^)	0.0015	0.0015
outer diameter (nm)	19.4	19.2
center-to-center dist. (nm)	19.4	20.5

**Table 2:** Estimated densities and scattering length density of different parts of HUB-4.

Part of the Molecule	Estimated Density ^a^ (g/cm^3^)	Scattering Length Density (× 10^10^ cm^−2^)
Amide chains ^b^	1.04	9.92
O–C_6_H_4_–COO– ^b^	1.55	19.63
–C_4_H_9_^c^	0.81	7.90
OC_6_H_4_COOC_4_H_9_	1.23	11.57

^a^ Estimated by incremental volumes [23]; ^b^ Values for crystalline packing; ^c^ Values for amorphous state.

## Supplementary Materials

The following are available online at https://www.mdpi.com/1422-0067/21/14/4960/s1. Figure S1: FTIR spectra of (a) Am-HU, (b) HUB-3 and (c) HUB-4, Figure S2: ^1^H-RMN spectra of (a) Am-HU, (b) HUB-3 and (c) HUB-4, Figure S3: ^13^C-RMN spectrum of Am-HU, Figure S4: ^13^C-RMN spectrum of HUB-3, Figure S5: ^13^C-RMN spectrum of HUB-4, Figure S6: Chromatograms of Am-HU, HUB-3 and HUB-4, Figure S7: ESI MS spectrum of Am-HU, Figure S8: ESI MS spectrum of HUB-3, Figure S9: ESI HRMS spectrum of HUB-3, Figure S10: ESI MS spectrum of HUB-4, Figure S11: ESI HRMS spectrum of HUB-4.

## Abbreviations

SAXS	Small-angle X-ray scattering
SANS	Small-angle neutron scattering
