# Supplementary material for: Structure of Nanotubes Self-Assembled from a Monoamide Organogelator
Source: Int J Mol Sci. 2020 Jul 14;21(14):4960. doi: 10.3390/ijms21144960 (PMC7404320; doi:10.3390/ijms21144960)
Supplement: Supplementary file 1 [file ijms-21-04960-s001.pdf]

Supplementary Materials

Structure of Nanotubes Self-Assembled from a Monoamide Organogelator

Samuel Zapién-Castillo [1], Nancy P. Díaz-Zavala [1,*], José A. Melo-Banda [1], Duncan Schwaller [2], Jean-Philippe Lamps [2], Marc Schmutz [2], Jérôme Combet [2] and Philippe J. Mésini [2,3,*]

[1] Centro de Investigación en Petroquímica, Tecnológico Nacional de México-Instituto Tecnológico de Ciudad Madero, Prolongación Bahía de Aldair, Ave. de las Bahías, Parque de la Pequeña y Mediana Industria, 89600 Altamira, México; samuel.zapien@iest.edu.mx (S.Z.-C.); aaron.melo@itcm.edu.mx (J.A.M.-B.)

[2] Université de Strasbourg, CNRS, Institut Charles Sadron, 23 rue du Loess, F-67000 Strasbourg, France; duncan.schwaller@etu.unistra.fr (D.S.); jean-philippe.lamps@ics-cnrs.unistra.fr (J.-P.L.); marc.schmutz@ics-cnrs.unistra.fr (M.S.); jerome.combet@ics-cnrs.unistra.fr (J.C.)

[3] International Center for Frontier Research in Chemistry, 8 allée Gaspard Monge, 67000 Strasbourg, France.

* Correspondence: nancy.dz@cdmadero.tecnm.mx (N.P.D.-Z.); mesini@ics-cnrs.unistra.fr (P.J.M.)

Content:

Figure S1. FTIR spectra of (a) Am-HU, (b) HUB-3 and (c) HUB-4.

Figure S2. ^{1}H-RMN spectra of (a) Am-HU, (b) HUB-3 and (c) HUB-4.

Figure S3. ^{13}C-RMN spectrum of Am-HU.

Figure S4. ^{13}C-RMN spectrum of HUB-3.

Figure S5. ^{13}C-RMN spectrum of HUB-4.

Figure S6. Chromatograms of Am-HU, HUB-3 and HUB-4.

Figure S7. ESI MS spectrum of Am-HU.

Figure S8. ESI MS spectrum of HUB-3.

Figure S9. ESI HRMS spectrum of HUB-3.

Figure S10. ESI MS spectrum of HUB-4.

Figure S11. ESI HRMS spectrum of HUB-4.

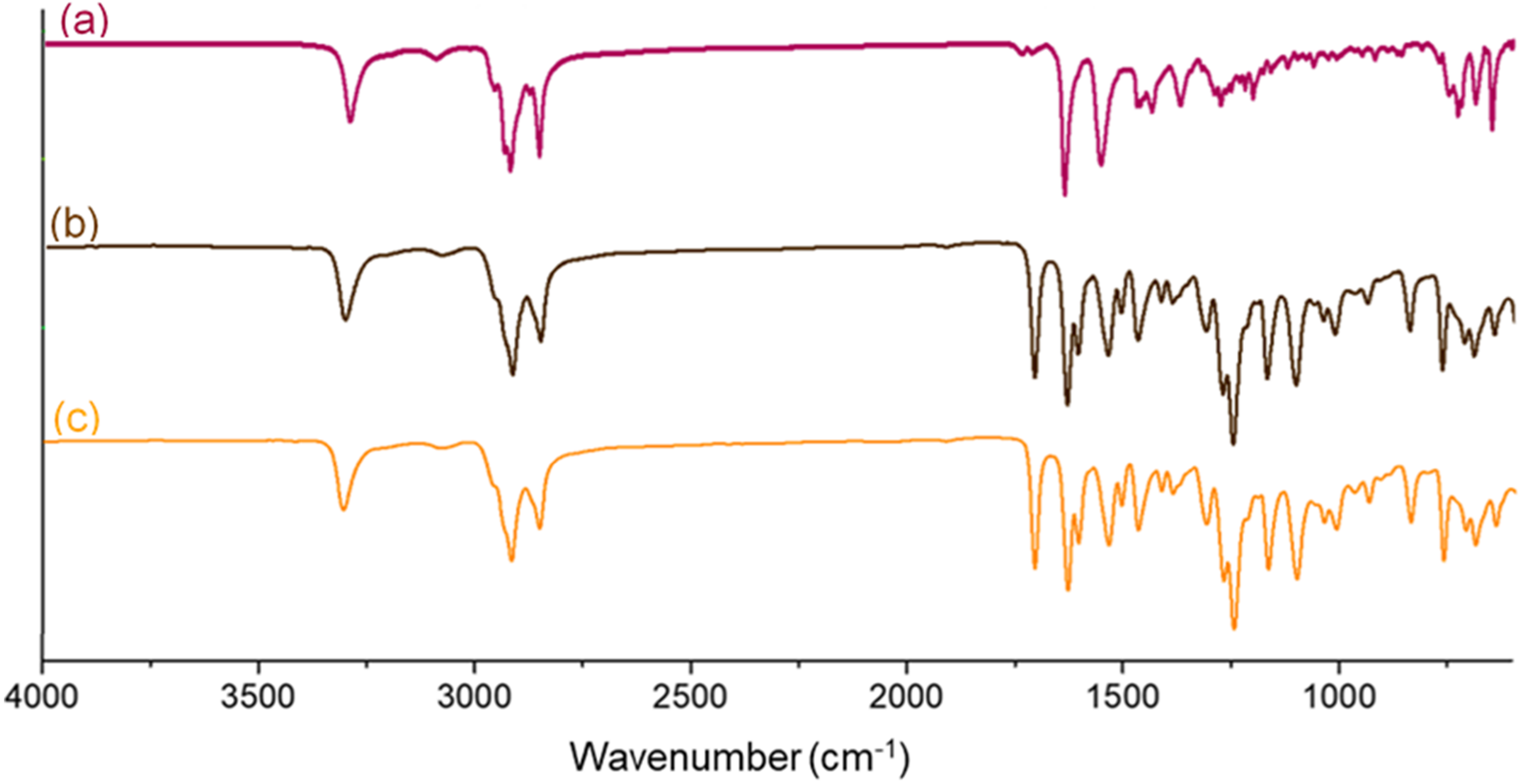

Figure S1. FTIR spectra of (a) Am-HU, (b) HUB-3 and (c) HUB-4.

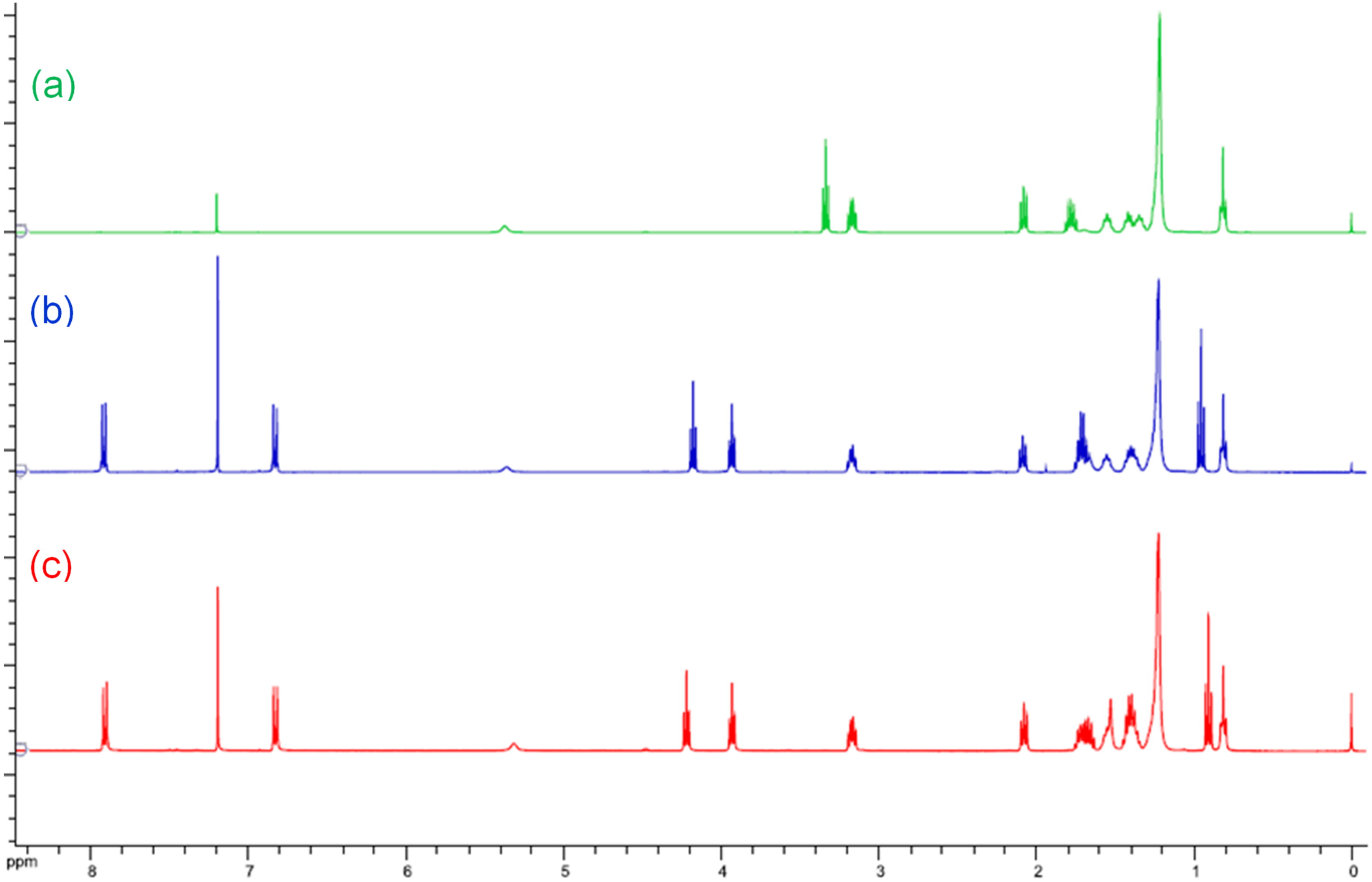

Figure S2. ^{1}H-RMN spectra of (a) Am-HU, (b) HUB-3 and (c) HUB-4.

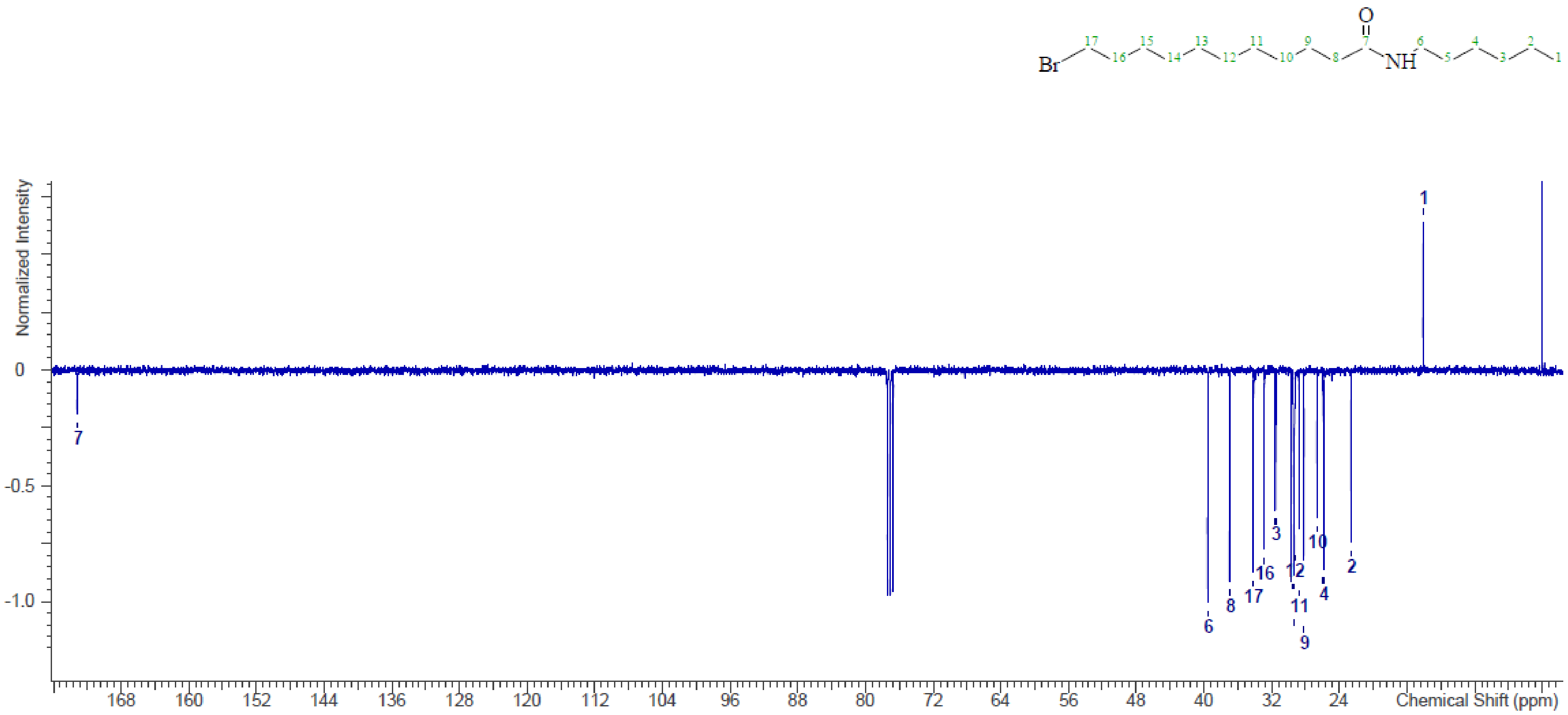

Figure S3. ^{13}C-RMN spectrum of Am-HU.

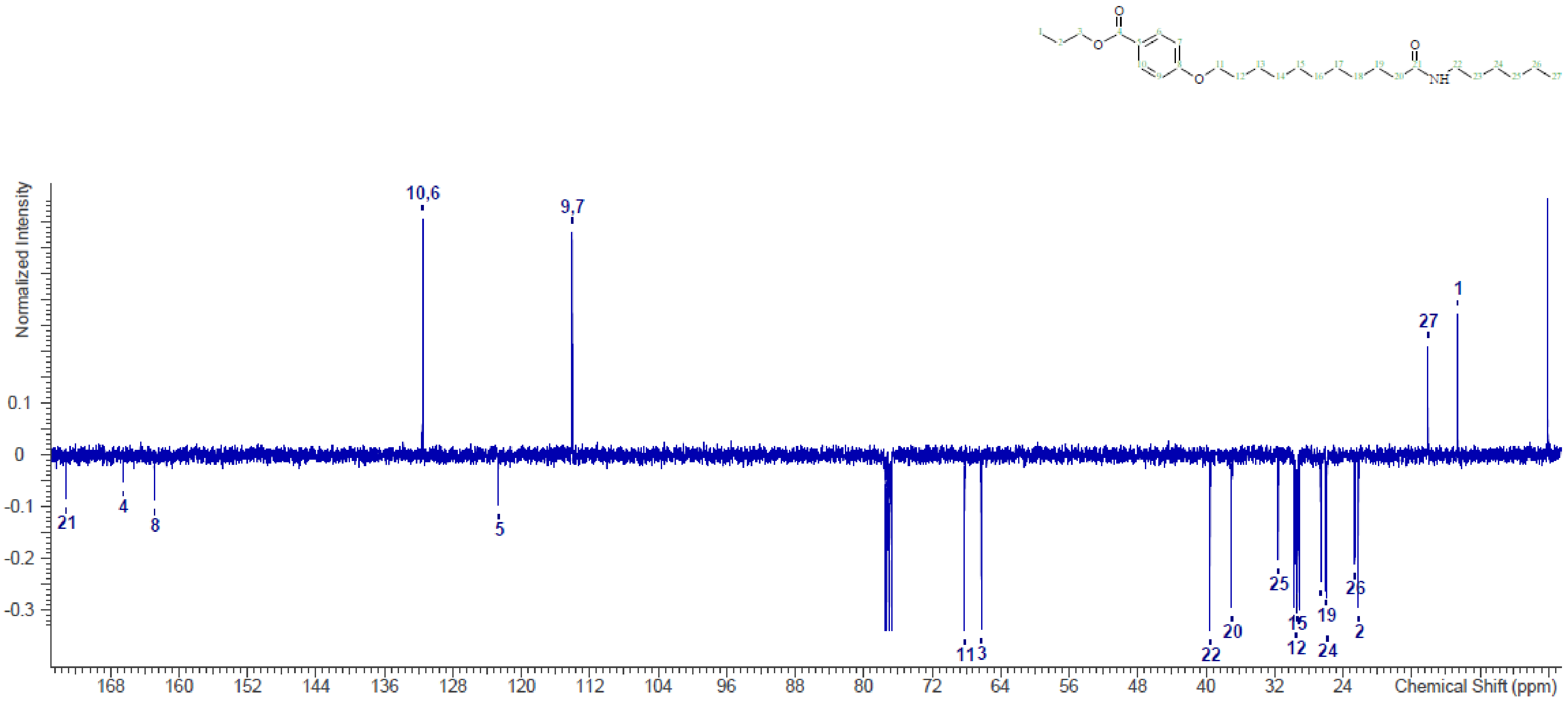

Figure S4. ^{13}C-RMN spectrum of HUB-3.

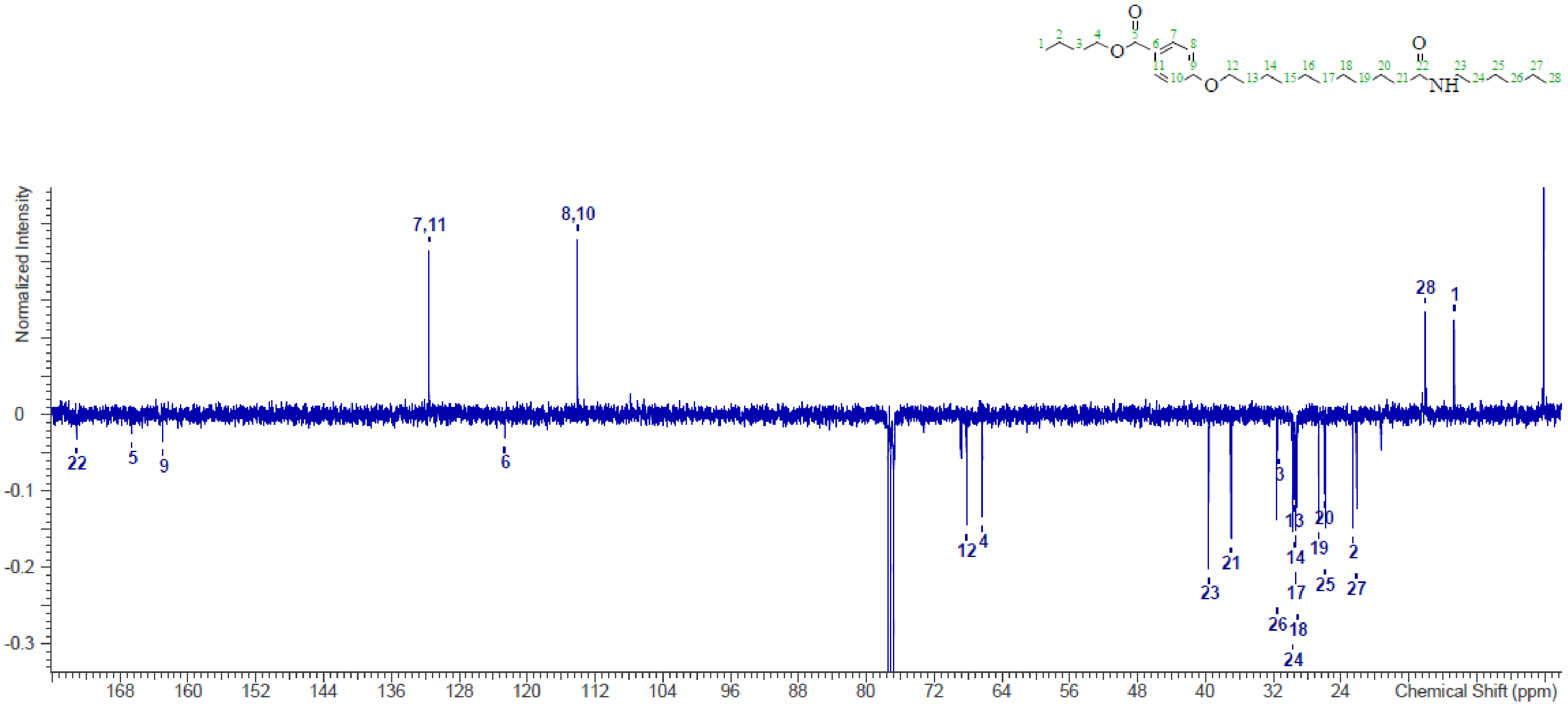

Figure S5. ^{13}C-RMN spectrum of HUB-4.

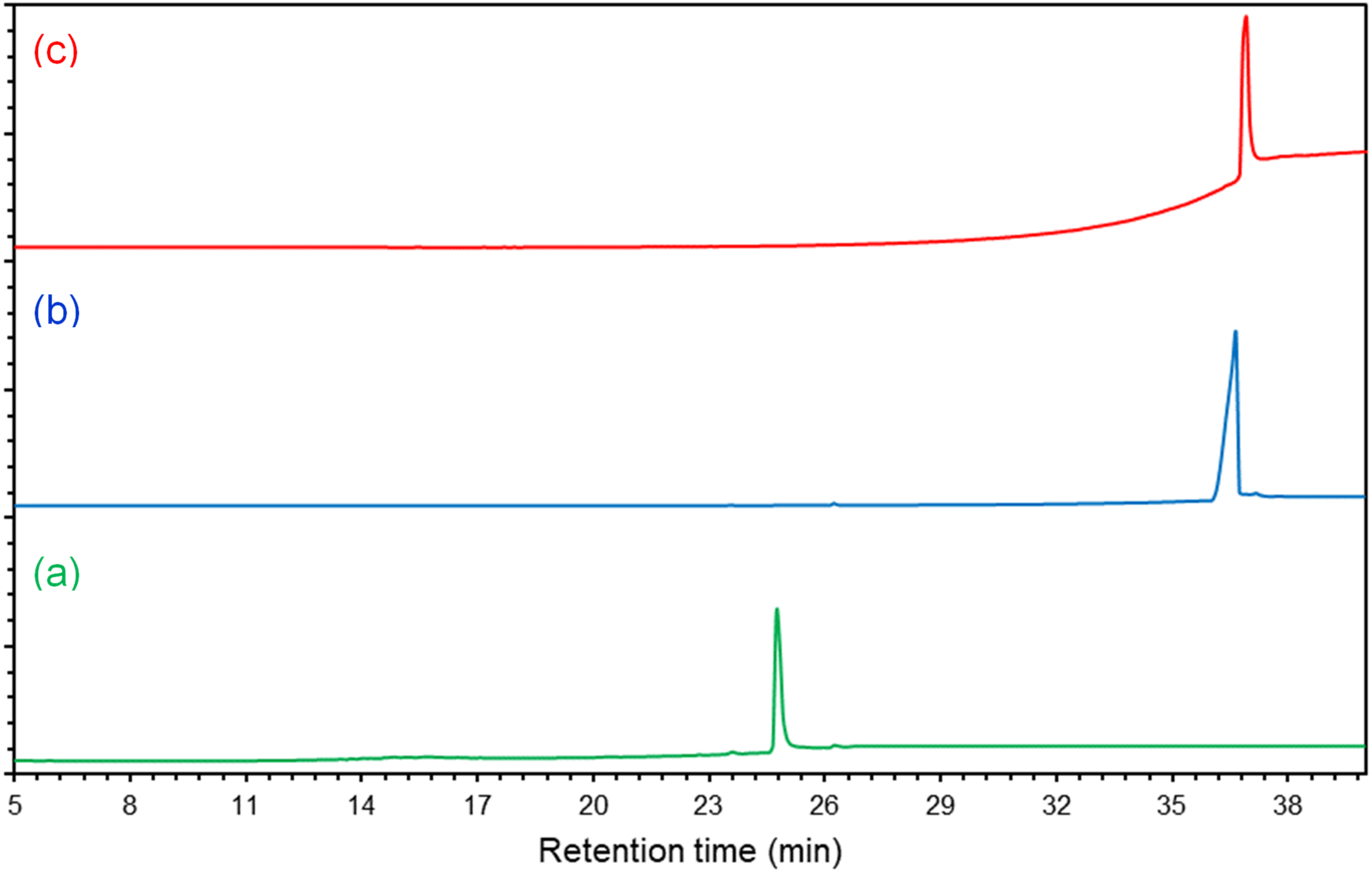

Figure S6. Chromatograms of (a) Am-HU, (b) HUB-3 and (c) HUB-4.

Acquisition Parameter

Source Type	ESI	Capillary	4500 V	Nebulizer	0.3 Bar	Set Hexapole RF	55.0 Vpp
Ion Polarity	Positive	Dry Heater	200 °C	Dry Gas	3.0 l/min	Set Capillary Exit	100.0 V

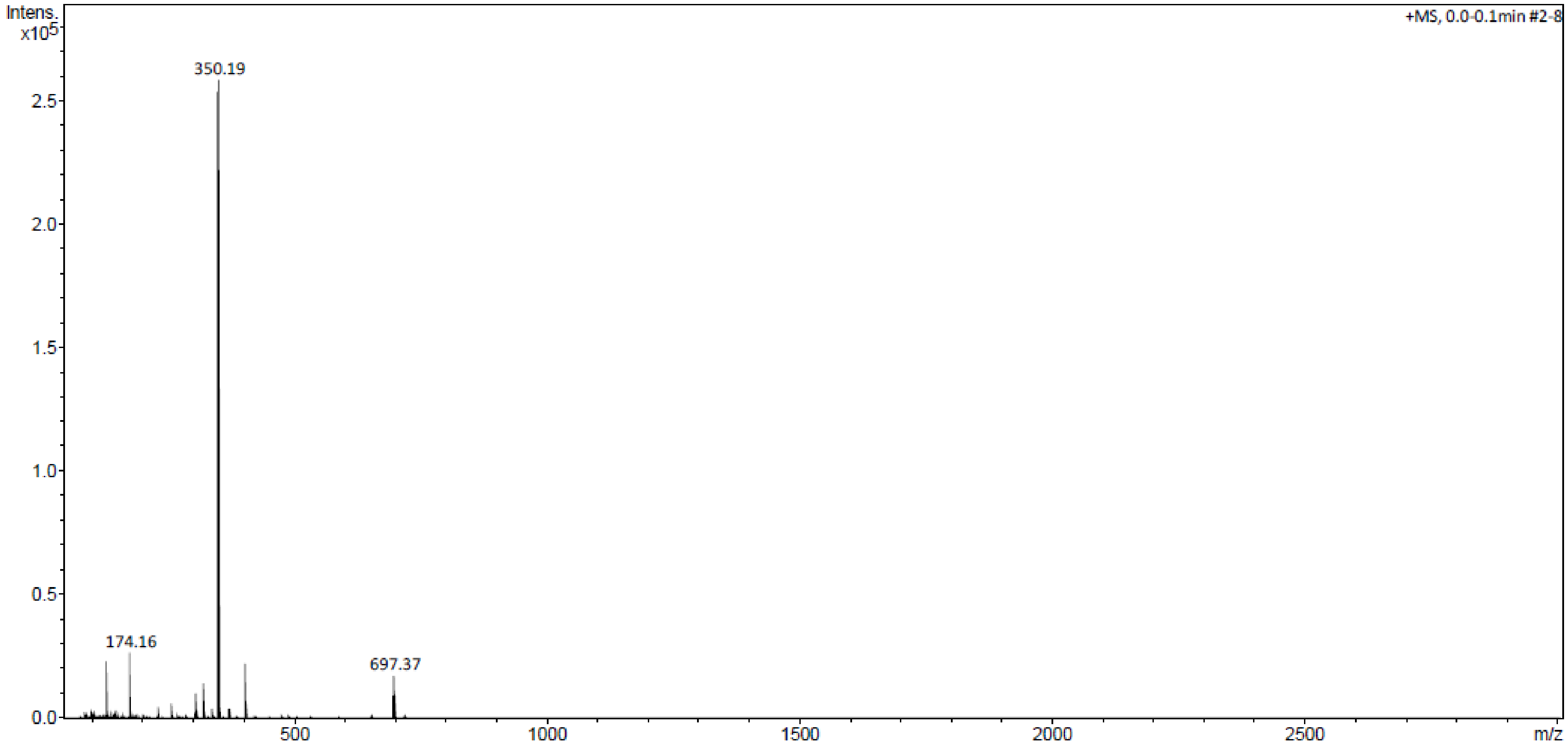

Figure S7. ESI MS spectrum of Am-HU.

Acquisition Parameter

Source Type	ESI	Capillary	4500 V	Nebulizer	0.3 Bar	Set Hexapole RF	55.0 Vpp
Ion Polarity	Positive	Dry Heater	200 °C	Dry Gas	3.0 l/min	Set Capillary Exit	100.0 V

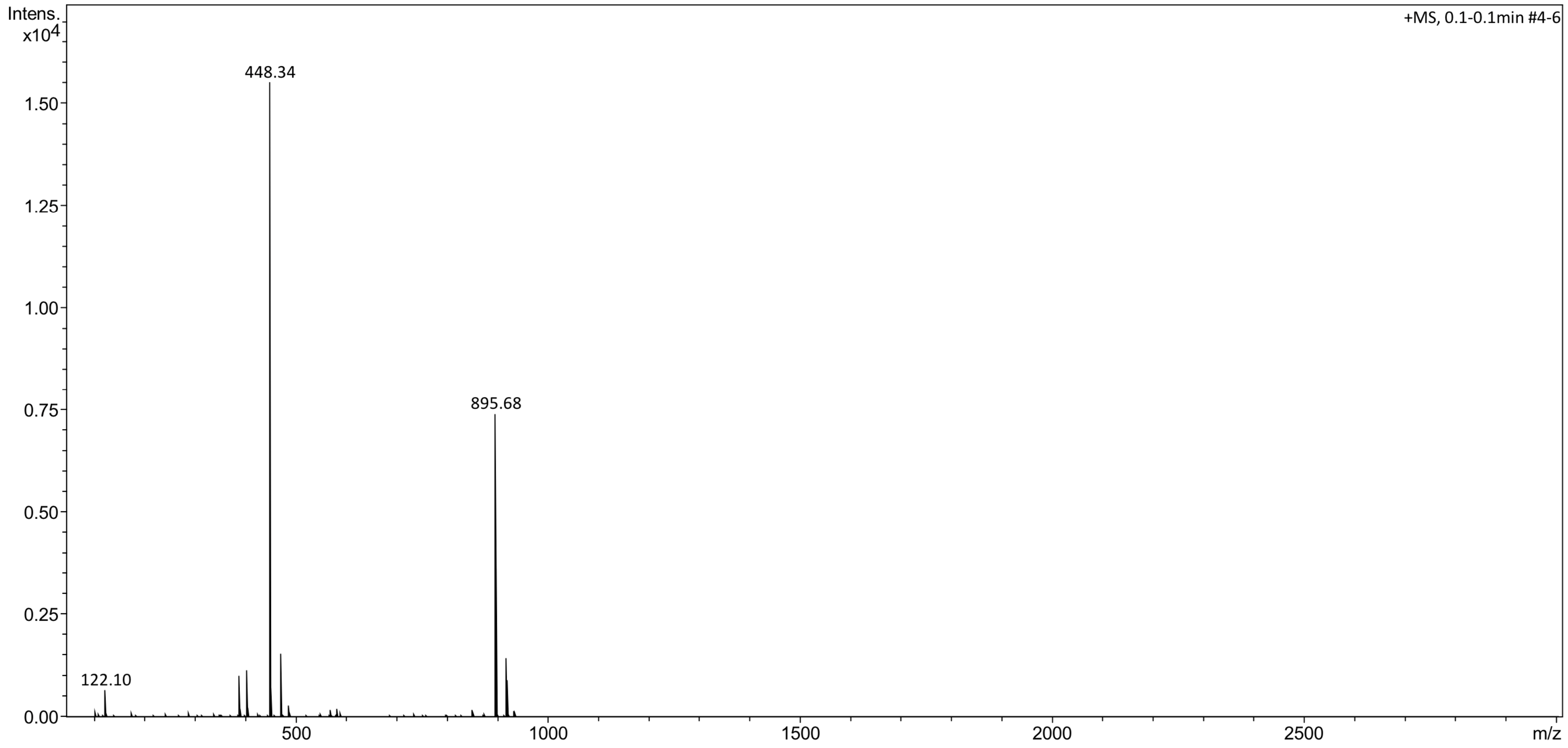

Figure S8. ESI MS spectrum of HUB-3.

Acquisition Parameter

				Set Corrector Fill	55.7 V
Source Type	ESI	Ion Polarity	Positive	n/a	n/a
n/a	n/a	n/a	n/a	n/a	n/a
Scan Begin	50 m/z	n/a	n/a	Set Reflector	1800.0 V
Scan End	3000 m/z	n/a	n/a	Set Flight Tube	8600.0 V
		n/a	n/a	Set Detector TOF	1961.2 V

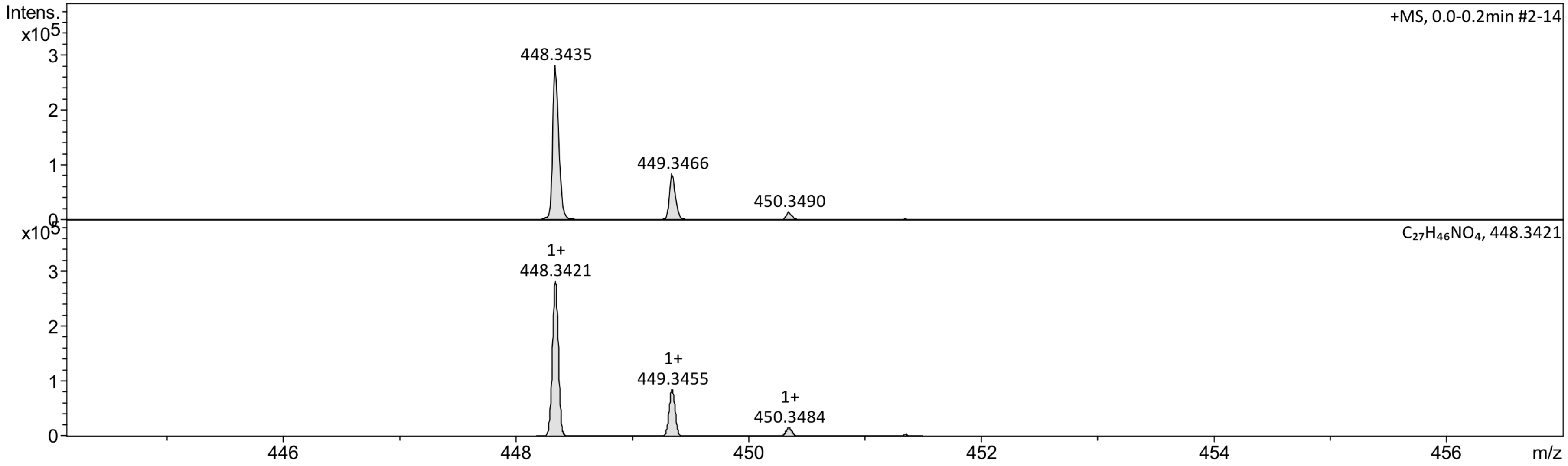

Meas. m/z	#	Ion Formula	m/z	err [ppm]	Mean err [ppm]	rdb	N-Rule	e^- Conf	mSigma	Std I	Std Mean m/z	Std I VarNorm	Std m/z Diff	Std Comb Dev
448.343469	1	C27H46NO4	448.342135	-3.0	-2.5	5.5	ok	even	3.9	5.6	n.a.	n.a.	n.a.	n.a.

Figure S9. ESI HRMS spectrum of HUB-3. (Top: experimental, bottom: simulated).

Acquisition Parameter

Source Type	ESI	Capillary	4500 V	Nebulizer	0.3 Bar	Set Hexapole RF	55.0 Vpp
Ion Polarity	Positive	Dry Heater	200 °C	Dry Gas	3.0 l/min	Set Capillary Exit	100.0 V

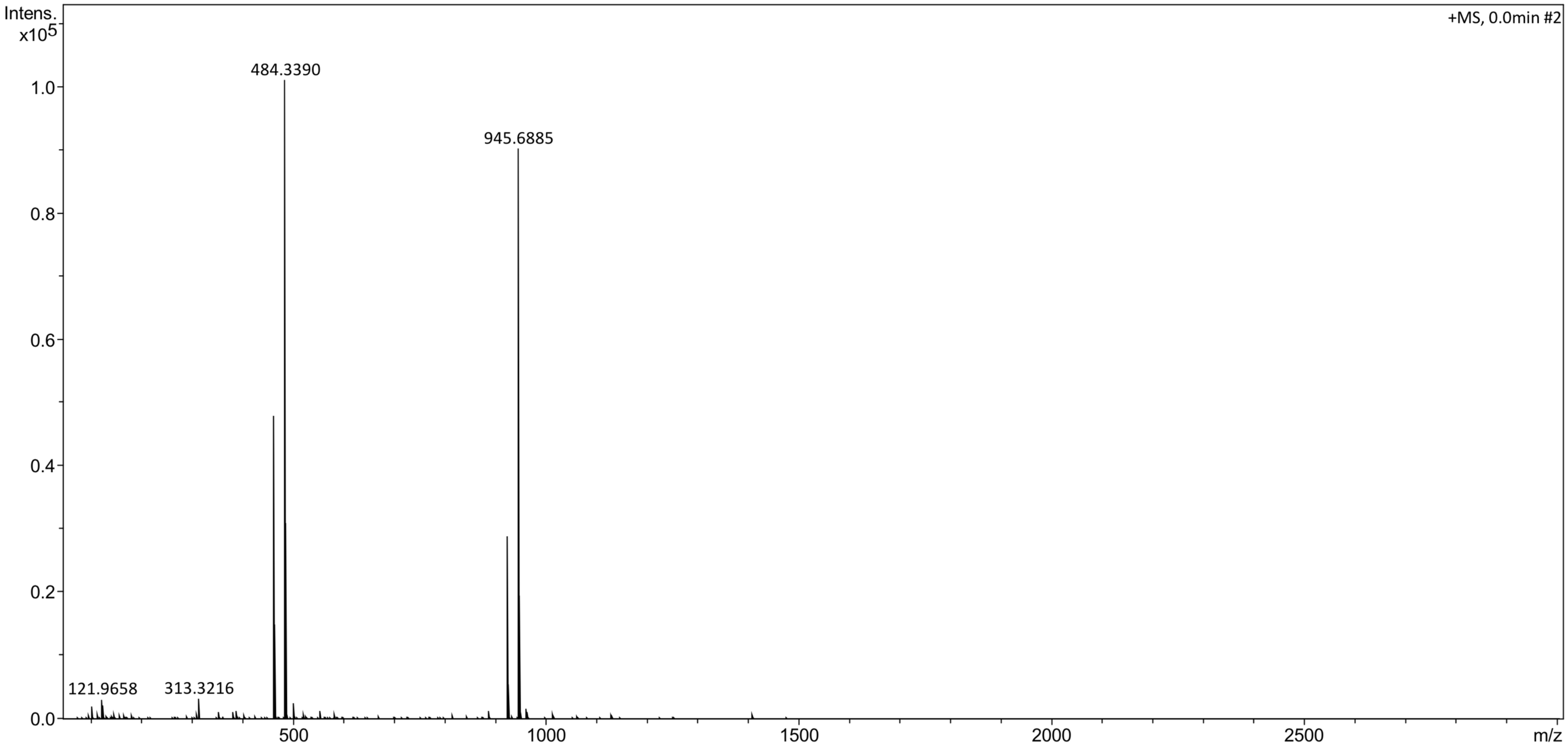

Figure S10. ESI MS spectrum of HUB-4.

Acquisition Parameter

				Set Corrector Fill	55.7 V
Source Type	ESI	Ion Polarity	Positive	n/a	n/a
n/a	n/a	n/a	n/a	n/a	n/a
Scan Begin	50 m/z	n/a	n/a	Set Reflector	1800.0 V
Scan End	3000 m/z	n/a	n/a	Set Flight Tube	8600.0 V
		n/a	n/a	Set Detector TOF	1961.2 V

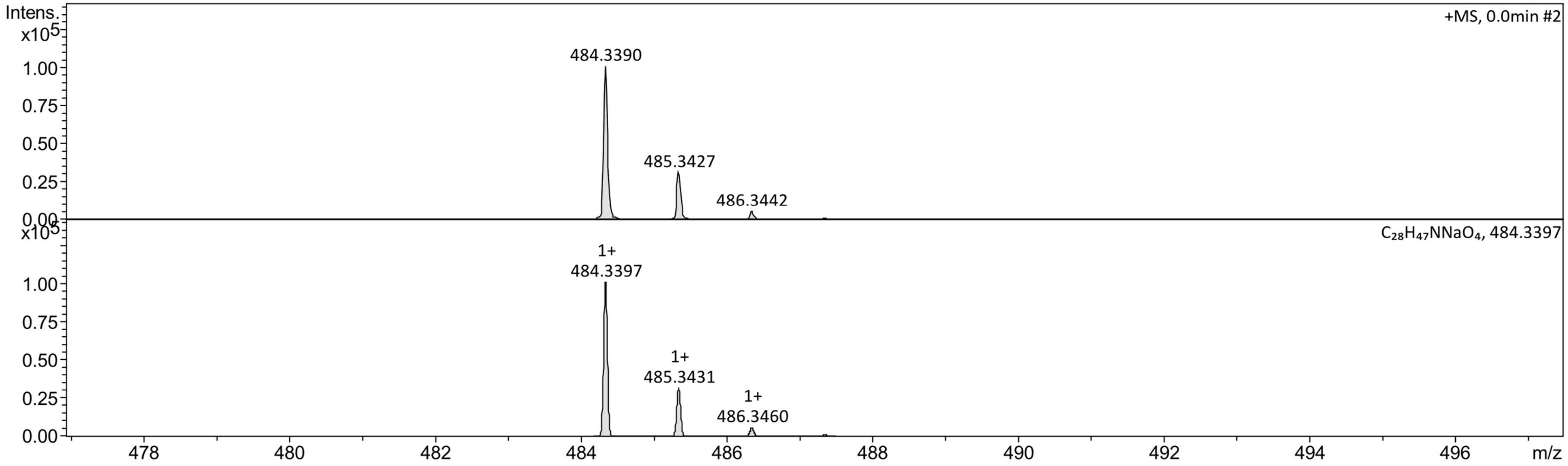

Meas. m/z	#	Ion Formula	m/z	err [ppm]	Mean err [ppm]	rdb	N-Rule	e¯ Conf	mSigma	Std I	Std Mean m/z	Std I VarNorm	Std m/z Diff	Std Comb Dev
484.338959	1	C28H47NNaO4	484.339730	1.6	2.2	5.5	ok	even	3.6	5.5	n.a.	n.a.	n.a.	n.a.

Figure S11. ESI HRMS spectrum of HUB-4 (Top: experimental, bottom: simulated).
